# Comparative Evaluation of Kit-M (GM-CSF and PGE-1) and Kit-I (GM-CSF and Picibanil) for the Generation of DCs/DCleus and Their Impact on Subsequent Antileukemic Immune Cell Activation Ex Vivo

**DOI:** 10.3390/cancers18172729

**Published:** 2026-08-23

**Authors:** Diana Deen, Olga Schutti, Tobias Baudrexler, Daniel Christoph Amberger, Caroline Plett, Lara Klauer, Joerg Schmohl, Peter Bojko, Doris Kraemer, Andreas Rank, Helga Schmetzer

**Affiliations:** 1 Department of Medicine III, University Hospital of Ludwig-Maximilian-University Munich, 81377 Munich, Germany; 2 Bavarian Cancer Research Center (BZKF), 86156 Augsburg, Germany; 3 First Department for Medicine, Paracelsus Medical University, 5020 Salzburg, Austria; 4 Department of Hematology, Diakonie Hospital, 70176 Stuttgart, Germany; 5 Department of Haematology and Oncology, Red Cross Hospital of Munich, 80634 Munich, Germany; 6 St. Josefs-Hospital, 58097 Hagen, Germany; 7 Department of Haematology and Oncology, University Hospital of Augsburg, 86156 Augsburg, Germany

**Keywords:** AML, leukemia-derived dendritic cells, DC/DCleu-generating protocols, antileukemic activity

## Abstract

**Background/Objectives**: Novel (immune) therapies are needed to stabilize the disease or achieve remissions in AML. We already demonstrated that DCleus can be generated ex vivo from AML patients’ blasts in WB using approved drugs (GM-CSF and PGE-1 (Kit-M) or GM-CSF and Picibanil (OK-432), Kit-I). The generated DCleus induce antileukemia-directed immune cells of the adaptive and innate immune system, enabling leukemia-specific immune activation after MLC. **Methods**: We compared the effects of Kit-I vs. Kit-M by quantifying (1) their potential to produce DCs/DCleus from WB samples from 6 healthy individuals and 28 AML patients’ WB in different stages of the disease and (2) the activation of adaptive and innate leukemia-specific IFNγ-producing or degranulating antileukemic cells after MLC with and without Kit-I/Kit-M-pretreated WB. Furthermore, we correlated the obtained results with the achieved improved cells’ antileukemic functionality and patients’ clinical data. **Results**: In AML samples we found significantly higher frequencies of (mature) DCleus generable without induction of blast proliferation in Kit-M as well as (although less pronounced) in Kit-I-treated vs. untreated samples, a significant increase in the frequency of (leukemia- specific) immunoreactive cells, and improved blast cytotoxicity after MLC with Kit-M and with Kit-I-treated vs. untreated samples (potentially with higher induction of CD4 and CIK IFNγ + cells in Kit-I-pretreated samples). These data might suggest that both Kits work via different pathways. **Conclusions**: We show that Kit-M and Kit-I produce DCs/DCleus and subsequently enhance (potentially via a different mode of action) antileukemic immune cell activation after MLC. Our findings point to a possibility to enhance antileukemic treatment in vivo by combining agents or identifying criteria for selecting an appropriate agent (combination) for the respective patient in the course of a personalized treatment strategy.

## Simple Summary

Novel (immune) therapies are needed to stabilize the disease or achieve remissions in acute myeloid leukemia. Using approved drugs, we are able to generate leukemia-specific dendritic cells (DCleus) that subsequently induce antileukemia-directed immune cells. We compared the effects of such drug combinations (Kit-I (containing granulocyte-macrophage colony-stimulating factor (GM-CSF) and Picibanil) vs. Kit-M (GM-CSF and Prostaglandin (PGE-1)) by quantifying (1) their potential to produce DCs/DCleus from leukemic whole blood (WB) and (2) their activation of leukemia-specific (IFNγ-producing or degranulating) or (3) blast-cytotoxic antileukemic cells. We found higher frequencies of DCleus without induction of blast proliferation, an increase in the frequency of (leukemia- specific) immunoreactive cells, and improved blast cytotoxicity in Kit-M- and Kit-I-pretreated vs. untreated WB after mixed lymphocyte culture (MLC): Kit-M generated comparable or slightly higher frequencies of DCs/DCleus compared to Kit-I and showed faster induction of blast cytotoxicity after MLC, as well as improved blast lysis compared to Kit-I. Our findings point to a possibility to enhance antileukemic treatment by combining agents or identifying criteria for selecting an appropriate agent (combination) for the respective patient.

## 1. Introduction

### 1.1. Current Therapy Strategies for Acute Myeloid Leukemia (AML)

Currently, treatment of AML is based on chemotherapy and, depending on the age and fitness of the patient, combined with allogeneic stem cell transplantation (SCT) [1]. However, about 80% of chemotherapy-treated and about 40% of SCT-treated patients relapse within two years [2,3,4]. There is a high need for maintenance therapies to stabilize disease or remissions. Different approaches aim to redirect the immune system in order to overcome leukemic immune escape and enforce a tumor-specific immune response. Promising options are targeted immunotherapies with antigen-presenting cells (APCs), such as dendritic cells (DCs) [5,6,7].

DCs are the most potent antigen-presenting cells; they link the innate and the adaptive immune system and are important initiators and regulators of an antigen-specific immune response [5,6,8,9]. While monocyte-derived DCs (moDCs) have to be loaded with leukemic antigens [5], DCleus can be converted directly from leukemic myeloid blasts, thereby presenting dendritic cells together with patient-specific leukemia antigens, e.g., [6,10]. Like moDCs, DCleus can be generated ex vivo without the need for loading with leukemic antigens and can subsequently be adoptively retransferred to patients. Both moDCs and DCleus have been shown to elicit a strong immune response mediated by leukemia-specific effector and memory cells ex vivo and in vivo, e.g., [5,11]. Several protocols for generating DCleus from leukemic WB (containing individual patients’ immune inhibitory or activating compounds) have been tested and evaluated. Combined response modifiers, such as Kit-M, containing GM-CSF and PGE1, or Kit-I, containing GM-CSF and Picibanil (OK-432), have been shown to produce mature DCleus without inducing blast proliferation. They give rise to immunoreactive effector and memory cells after MLC that improve antileukemic cytotoxicity, independent of patients’ age, sex, risk categorization, HLA, or transplantation status [6,7,10,12]. Preliminary data showed that, among many Kits tested in parallel, Kit-M was the superior Kit to produce DCleu and DCleu-mediated antileukemic reactions. As shown after treatment of three refractory AML patients (before or after SCT), this (DC-mediated) immune activation can also be induced after Kit-M treatment in vivo [11,13]. In some preliminary settings, Kit-M and Kit-I were shown to induce antileukemic reactions ex vivo and even in vivo in a rat model, e.g., [6,11]. With the study presented here, we aimed to systematically compare ex vivo-induced effects on the generation of DCleus, as well as antileukemic effects triggered by Kit-M vs. Kit-I, and to correlate findings with ex vivo-achieved and patients’ clinical data in order to detect samples with potentially inducible superior Kit-I or Kit-M antileukemic reactions.

A challenge in the future could be (after selection of the better Kit ex vivo) to treat AML patients with Kit-M or Kit-I, thereby enabling the patient to produce (DC/DCleu-activated) leukemia-specific effector or memory cells in vivo, which eliminate blasts and stabilize remissions by memory cell activity.

### 1.2. Innate and Adaptive Immune-System-Mediated Leukemia-Specific Response

To attack pathogens and tumor cells, the innate and adaptive immune systems use humoral and cellular mechanisms, respectively [10]. The innate immune system, consisting of macrophages (CD15+), monocytes (CD14+), DCs (CD80+CD206+, etc.), cytokine-induced killer cells (CIK, CD3+CD56+), and natural killer cells (NK, CD3−CD56+), is responsible for the ‘first-line’ response. The adaptive immune system is responsible for a specific and ‘long-term’ immunity, with key players such as B cells (CD19+), T cells (CD3+), and their subpopulations such as non-naive T cells (CD3+CD4+ or CD4−CD45RO+), CD4+CD3+ (TCD4+), and CD4−CD3+ (TCD4−) as ‘active’ mediators of immune responses and central memory cells (CD3+CD45RO+CD197+: Tcm) or effector-memory cells (CD3+ CD45RO+ CD197−: Tem) to fight reoccurring tumor cells [10] (see Table 1 for details).

To detect and quantify leukemia-specific IFNγ- and TNFα-producing (leukemia antigen-specific) cells, we applied the intracellular cytokine assay (INCYT), as well as the Cytokine Secretion Assay (CSA) and the degranulation (DEG) assay, to monitor lysosomally associated membrane glycoproteins (LAMPs), CD107a+ cells that are involved in perforin-associated degranulation processes [6]. The subtypes of leukemia-specific innate and adaptive (effector and memory) cells are given in Table 1. With the cytotoxicity assay (CTX), the cytotoxic effect of effector cells against target cells can be determined. We were thereby able to quantify the antileukemic effect of (induced) effector cells against blasts after the influence of stimulator cells (e.g., Kit-treated vs. untreated WB) in MLC [10,12].

### 1.3. Aims of This Study

The aim of this study was to (1) characterize uncultured immune cells from AML patients and healthy donors by evaluating the qualitative and quantitative composition of (leukemia) specific innate and adaptive immune cells, (2) evaluate the potential of Kit-I (vs. Kit-M) to produce DCs/DCleus from AML patients’ WB, (3) evaluate the activation of adaptive and innate antileukemic cells after MLC (stimulated with Kit-I vs. Kit-M), (4) compare effects of Kit-I vs. Kit-M on improved (leukemia-specific, blast-lytic) activation of immune cells and provision of (leukemia-specific) memory cells and effects on blast lysis, (5) correlate results with the achieved improved cells’ antileukemic functionality and patients’ clinical data, (6) detect samples with potentially inducible superior Kit-I or Kit-M antileukemic reactions, and to deduce the ‘best’ treatment approach.

## 2. Materials and Methods

### 2.1. Sample Acquisition and Preparation

Sample collection of whole blood (WB) was conducted after obtaining the written informed consent of blood donors in accordance with the Declaration of Helsinki and the ethical committee of the LMU in Munich (No. 33905). The heparinized peripheral WB samples and clinical reports were provided by the University Hospitals of Augsburg, Oldenburg, and Munich, as well as the Diakonieklinikum Stuttgart, the Rotkreuzklinikum Munich, and the St. Josefs Hospital Hagen.

Blood samples were collected from 28 AML patients and 6 healthy volunteers. The patients were, on average, 57.9 (range 21–83) years old on the day of sample collection; healthy individuals were 31.5 (range 22–54) years old. For patients, the female-to-male ratio was 1:1.8 and 1:5 for the healthy ones. The patients were classified according to the French–American–British (FAB) classification [15], the etiology (primary and secondary AML), the stage of the disease (first diagnosis, relapse), the blast phenotype, blast frequencies in peripheral blood, and the European Leukemia Net (ELN) risk classification [2]. Patients’ peripheral WB contained, on average, 28.3 (range 1–65) % blasts, as measured by flow cytometry. Patients’ characteristics are given in Table 2.

The cellular composition of WB samples from AML patients and healthy volunteers was evaluated in uncultured WB as well as after dendritic cell culture (DCC) and T-cell- enriched mixed lymphocyte culture (MLC) by flow cytometry. Potentially antigen- or leukemia-specific cells were quantified using the DEG and INCYT or CSA assays in patients’/healthy donors’ WB samples. DCs were cultured from WB. MNCs were isolated using Ficoll–Hypaque density gradient centrifugation. T cells were then isolated from MNCs via MACS MicroBead Technology (Miltenyi Biotec, Bergisch Gladbach, Germany) and frozen for later use. Frozen samples were stored at −80 °C until utilization. All procedures were performed as described before [10,11].

### 2.2. Characterization and Quantification of Cells via Flow Cytometry

To quantify frequencies and phenotypes of cell subpopulations of leukemic blasts, DCs, monocytes, B, T, NK, and CIK cells and to assess their (leukemia-specific) functionality, flow cytometric analyses were performed as described before [10]. These analyses were performed before and after cell culture. Abbreviations for all cell types and surface markers used are given in Table 1. For cell staining, various monoclonal antibodies (moAbs) labeled with Fluorescein Isothiocyanate (FITC), Phycoerythrin (PE), Phycoerythrin Cyanine 7 (PC7), or Allophycocyanin (APC) were used. 7-AAD was used to detect non-viable cells. To quantify degranulating cells, a FITC-conjugated moAb against CD107a was used. To quantify intracellular cytokine (IFNγ and TNFα) producing cells or to detect IPO38-positive cells, fixation and permeabilization were conducted using Medium A and Medium B (FiX&PERM, Thermo Fisher Scientific, Waltham, MA, USA). All flow cytometric measurements were conducted using a FACSCalibur flow cytometer (Becton Dickinson, Macquarie Park, NSW, Australia) and the analysis software CellQuestPro (Becton Dickinson, version 5.1), using a uniformly defined gating strategy as described before [6,10].

### 2.3. Dendritic Cell Culture (DCC) and Mixed Lymphocyte Culture (MLC)

DCs and DCleus were generated by treating patients’or healthy donors’ WB with Kit-I or Kit-M as described before [6]. DCs/DCleus containing Kit-M- or Kit-I-pretreated or not-pretreated WB samples were then used as stimulators to activate T-cell-enriched immune cells in MLC as described before [10]. Flow cytometric analyses of different immune cell subtypes were performed before and after MLC [6,10].

### 2.4. Detection of Antigen-Specific Cells Using Intracellular Cytokine Assay (INCYT), Cytokine Secretion Assay (CSA), and Degranulation Assay (DEG)

(Intracellularly) different IFNγ- or TNFα-producing immune cells were quantified by INCYT and CSA (IFNγ). These cytokine assays were performed in uncultured healthy and leukemic WB and leukemic WB after MLC, with and without Kit-I or Kit-M stimulation, as described before [6,10]. Additional stimulation of potentially leukemia-specific cells before culture was achieved by adding leukemia-associated antigens (LAAs): “Wilms Tumor 1” (PepTivator^®^WT1, Miltenyi Biotec) and “Preferentially Expressed Antigen of Melanoma” (PepTivator^®^PRAME, Miltenyi Biotec). Healthy WB samples were stimulated with 10 µg/mL staphylococcal enterotoxin B (SEB, Sigma-Aldrich, St. Louis, MO, USA) to demonstrate potentially antigen-specific cell activation and the reliability of antigen-specific tests. To avoid loss or weakening of CD107a antibodies’ fluorescence, a 2 μg/mL Monensin solution (BioLegend, San Diego, CA, USA) was added to the cultures. Cells were analyzed by flow cytometry as described before [10,16].

As a marker for induced cell cytotoxicity, degranulating cells were quantified using a FITC-conjugated antibody against CD107a as described before [10]. Assays were performed in uncultured healthy or leukemic WB as well as after DCC (Control)/(Kit-I or Kit-M).

### 2.5. Cytotoxicity Fluorolysis Assay (CTX)

To investigate the ability of effector cells to lyse target cells, a cytotoxicity fluorolysis assay (CTX) was conducted. T-cell-enriched cells were stimulated with Kit-I- or Kit-M- treated WB after DCC (Control)/(Kit-I or Kit-M) (‘effector cells’). As ‘target cells’, thawed viable patients’ blasts containing MNCs were used. Effector and target cells were co-cultured for 3 and 24 h as described before [10]. Target cells were stained with the respective blast antibodies (see Table 1) and analyzed by flow cytometry [10].

The lytic activity against blasts (“blast lysis”) was defined as the difference in frequencies of viable blasts in the effector–target-cell cultures compared to controls, and “improved blast lysis” was defined as the difference in proportions of “blast lysis” achieved in DCC (Kit-I or Kit-M) compared to DCC (Control) [10].

An experimental workflow for all experiments is given in Supplementary Figure S1.

### 2.6. Statistical Methods

The data obtained from flow cytometry were further processed with Excel (Microsoft, version 2404, Redmond, WA, USA) and PRISM 9 (GraphPad Software, Software version number 9.5.1, San Diego, CA, USA). Data are presented as mean ± standard deviation. Statistical comparisons of two groups were performed using an independent-samples two-tailed *t*-test. The strength of the relationship between two variables was classified as: *p*-values > 0.1 = “not significant”; *p*-values between 0.1 and 0.05 = “numerical trend”; *p*-values ≤ 0.05 = “significant”; *p*-values ≤ 0.005 = “highly significant”.

## 3. Results

### 3.1. Prologue

We analysed the composition of (antigen-specific) immune cells in uncultured AML patients compared to healthy WB samples. We compared the efficacy of Kit-I (vs. Kit-M) to generate DCs/DCleus from AML and healthy WB samples and of Kit-I (vs. Kit-M) pretreated AML and healthy WB samples to activate antigen/antileukemia-specific immune effector and memory cells using DEG and INCYT/CSA assays and analysed their efficacy to improve antileukemic cytotoxicity using a CTX Assay.

### 3.2. Composition of (Antigen-Specific) Immune Cells in Uncultured Healthy and AML WB Samples

**Figure 1:**
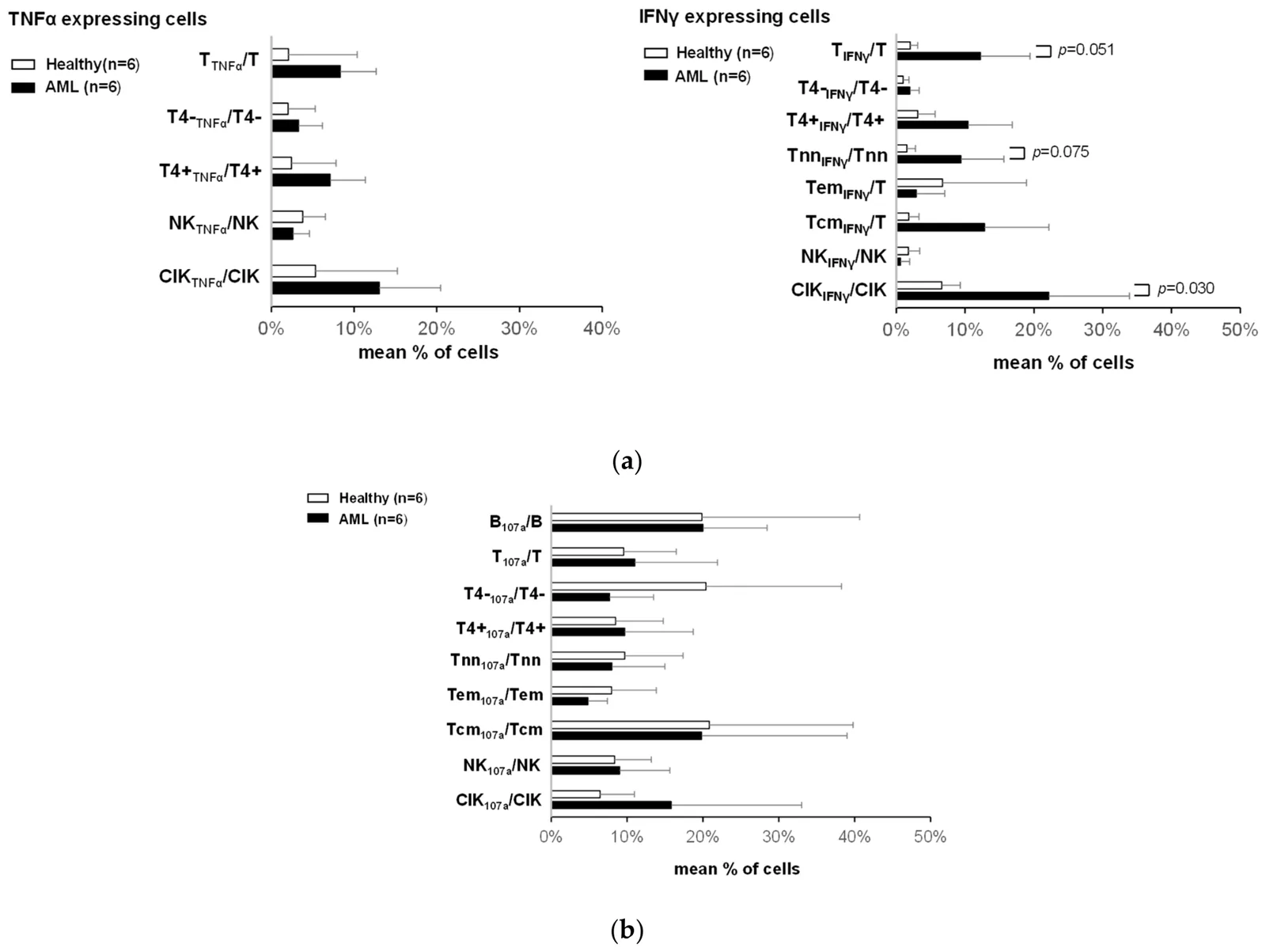
Composition of (antigen-specific) immune cells in uncultured healthy and AML WB samples. (**a**) The composition of TNFα- and IFNγ-producing/secreting cells in uncultured WB after stimulation with LAA/SEB and (**b**) the composition of degranulating cells in uncultured WB after stimulation are given. Cytokine-producing cells comprise intracellularly producing and secreting cells. IFNγ- or TNFα-producing cells can be detected by intracellular cytokine assay (INCYT) and IFNγ-secreting cells by Cytokine Secretion Assay (CSA). Given are the mean frequencies ± standard deviation (SD) of T/B cells and innate immune cells. Statistical significance was tested by an independent-samples two-tailed *t*-test. (n) number of cases; significance is defined as “highly significant” in cases with *p*-values ≤ 0.005, “significant” with *p*-values ≤ 0.05, a “numerical trend” with *p*-values between 0.05 and 0.1, and “not significant” with *p*-values ≥ 0.1. Abbreviations of cell subpopulations are given in Table 1.

We found higher frequencies of most cytokine-producing immune cells (as detected with INCYT or CSA assay after stimulation with LAA/SEB) in AML patients’ (n = 6) vs. healthy (n = 6) WB samples (e.g., %T_TNFα_/T (AML) 8.33 ± 5.34; %T_TNFα_/T (Healthy) 2.05 ± 1.37; %Tnn_IFNγ_/Tnn (AML) 9.40 ± 8.49; %T_IFNγ_/Tnn (Healthy) 1.54 ± 1.24; *p* = 0.051; Figure 1a). Data on CSA and INCYT-IFNγ-detected cells were pooled, since in preliminary experiments data have been shown to be comparable [6]. We found comparable or lower frequencies of antigen-specific (cytokine-producing or degranulating) cells in healthy samples after SEB and in leukemic samples after WT1/PRAME stimulation (pointing to the activatability of antigen-specific cells in both cohorts): frequencies of CD107a^+^ cell subsets in AML patients’ vs. healthy WB samples (e.g., %Tem_107a_/Tem (AML) 4.83 ± 2.56; %Tem_107a_/Tem (Healthy) 7.95 ± 5.90; Figure 1b).

### 3.3. Effects of Kit-M and Kit-I on the Generation of DCs/DCleus and Blast Proliferation from AML and Healthy WB Samples

**Figure 2:**
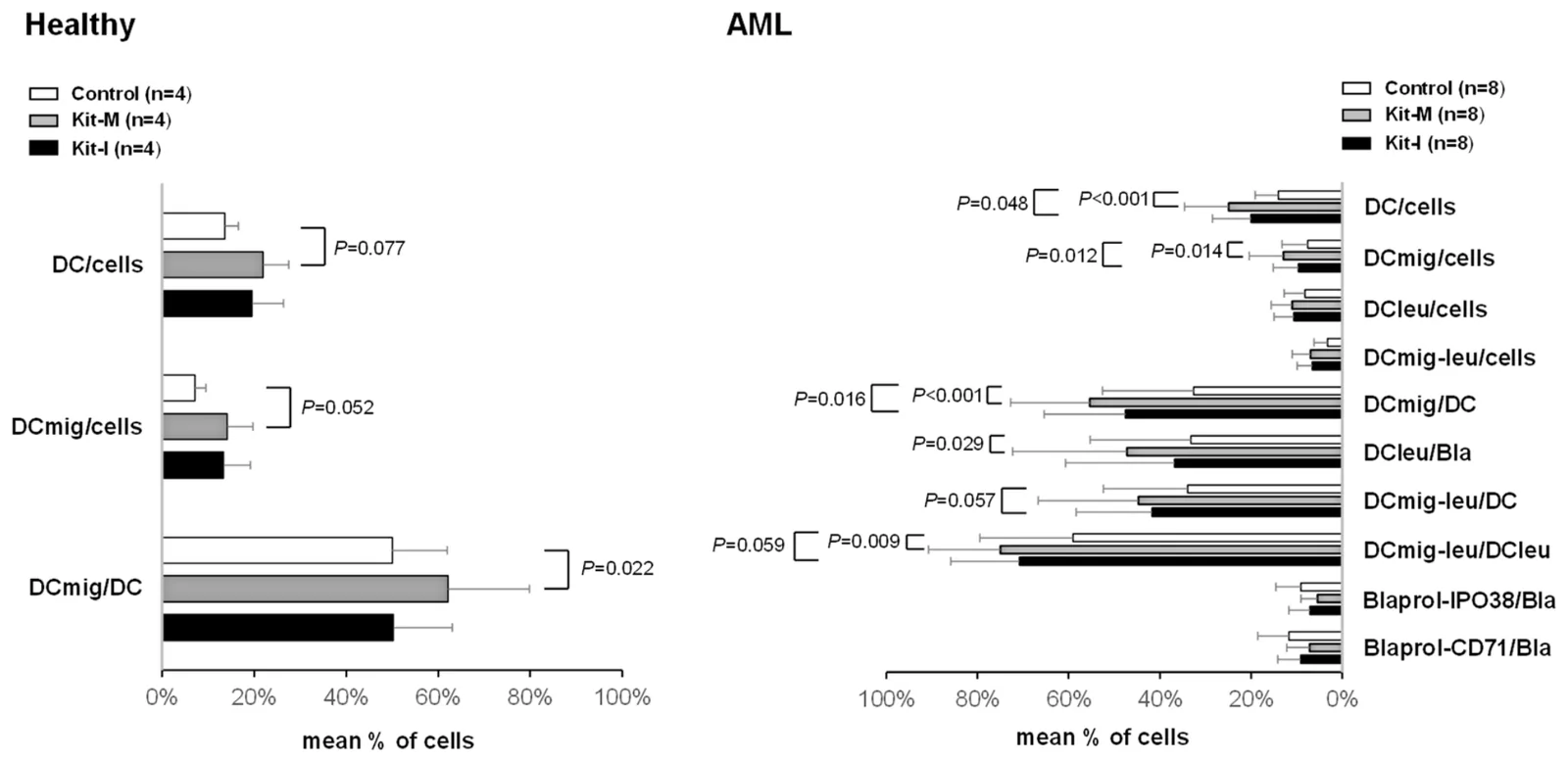
Effects of Kit-M- and Kit-I-treated AML and healthy WB on the generation of DCs/DCleus and blast proliferation. Shown are DC subtypes in healthy and leukemic WB cells after 7 d incubation with Kit-I vs. Kit-M vs. control. Given are the mean frequencies ± standard deviation (SD) of cell subsets. Statistical significance was tested by an independent-samples two-tailed t-test. (n) number of cases; significance is defined as “highly significant” in cases with *p*-values ≤ 0.005, “significant” with *p*-values ≤ 0.05, a “numerical trend” with *p*-values between 0.05 and 0.1, and “not significant” with *p*-values ≥ 0.1. Abbreviations of cell subpopulations are given in Table 1.

Compared to cultures without added Kits, we found significantly increased frequencies of mature DCs and DCleus under the influence of Kit-I and Kit-M without induction of blast proliferation (e.g., %DCleu/cells (Kit-I) 10.60 ± 4.34; %DCleu/cells (Kit-M) 11.02 ± 4.56; %DCleu/cells (Control) 8.15 ± 4.59; Figure 2). Comparing the efficacy of Kit-M or Kit-I vs. control to produce DCs and DCleus in samples that were analyzed in parallel (n = 4 in Healthy; n = 8 in AML samples), we found significantly higher frequencies of mature DCs or DCleus in samples treated with either Kit-I or Kit-M compared to control (e.g., %DCmig/cells (Kit-M): 12.93 ± 7.44, %DCmig/cells (Control): 7.50 ± 5.70, *p* = 0.014, and %DCmig/cells (Kit-I): 12.93 ± 7.44, %DCmig/cells (Control): 7.50 ± 5.70, *p* = 0.012; Figure 2). We found comparable or higher frequencies of mature DCs or DCleus in samples treated with Kit-M vs. samples treated with Kit-I (e.g., %DCmig/DC (Kit-M): 55.04 ± 17.32; %DCmig/DC (Kit-I): 47.48 ± 17.91; Figure 2).

Using WB of healthy volunteers, we found comparable or higher frequencies of DC and DCmig in the Kit-treated cultures vs. controls. Due to small sample numbers, we found a numerical trend toward higher frequencies of DCs or DCmig only in samples treated with Kit-M (e.g., %DCmig/cells (Kit-M): 14.15 ± 5.60, %DCmig/cells (Control): 7.16 ± 2.36, *p* = 0.052, and %DCmig/cells (Kit-I): 13.38 ± 5.80, %DCmig/cells (Control): 7.16 ± 2.36; Figure 2).

### 3.4. Effects of Kit-M- or Kit-I-Pretreated WB on the Provision of Activated Immune Cells in AML Samples or Antigen-Specific Immune Cells in AML and Healthy Samples After MLC

**Figure 3:**
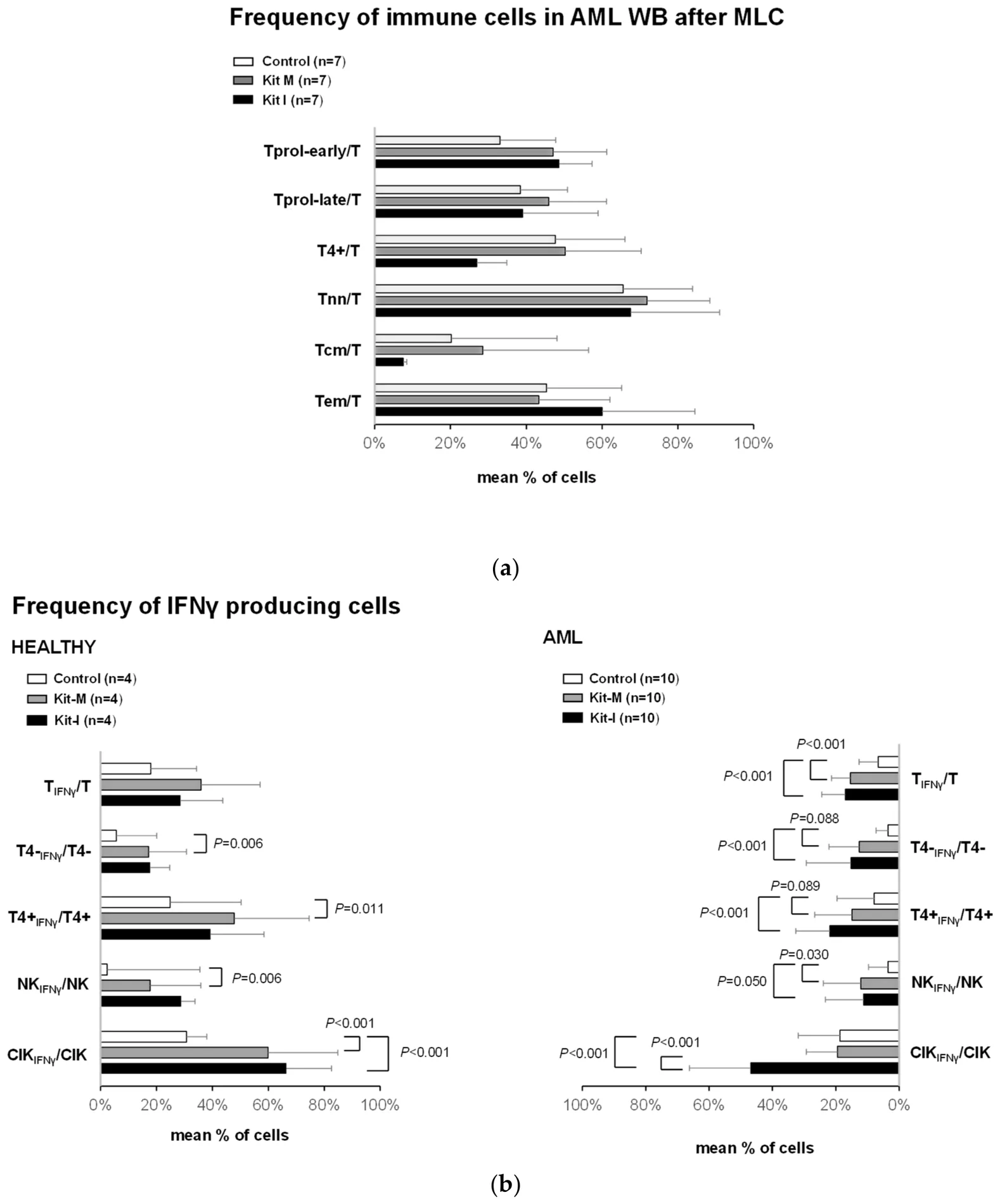

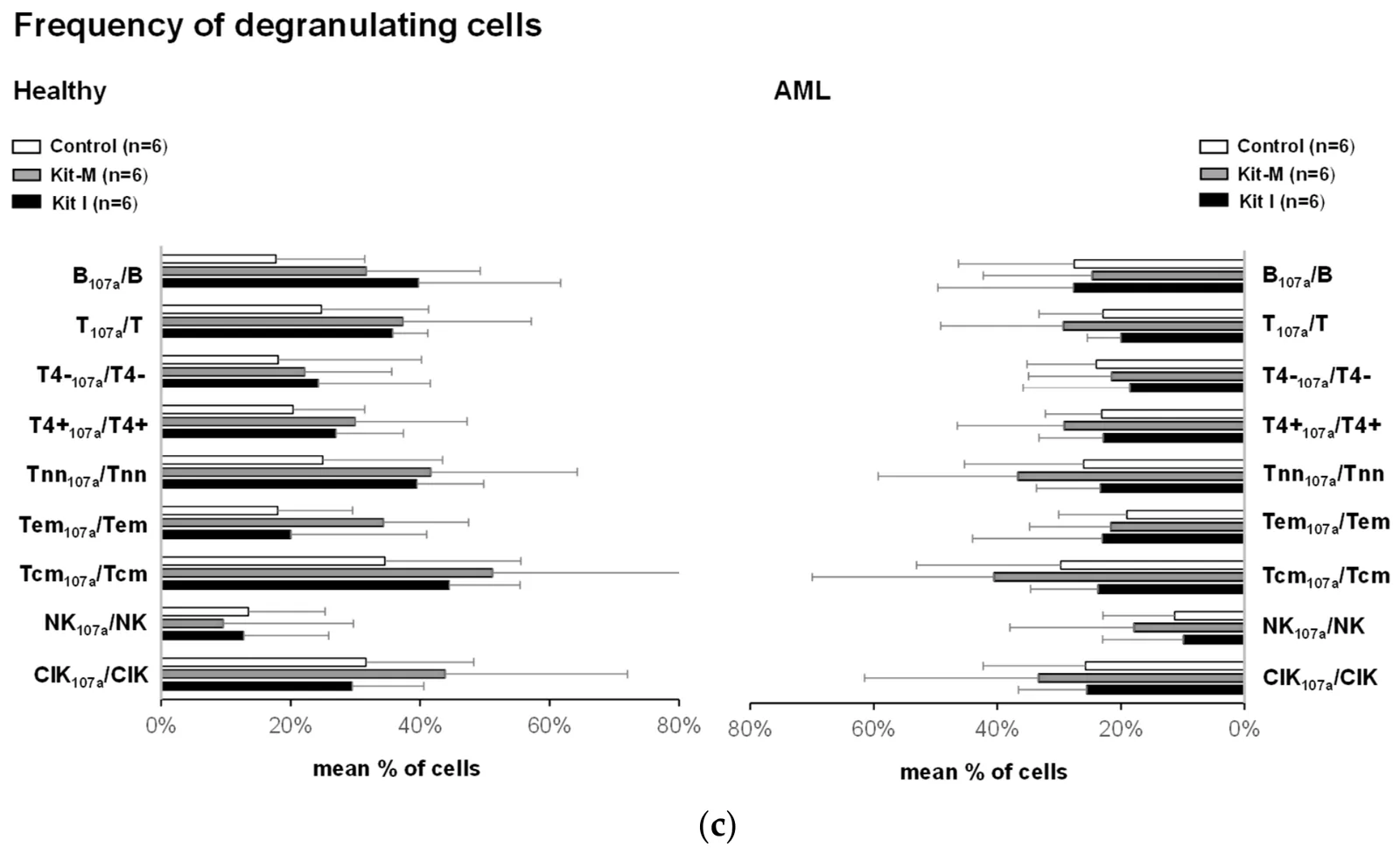
Effects of Kit-M- and Kit-I-treated AML or healthy WB on the provision of activated / antigen-specific immune cells in AML or healthy samples after MLC culture. Samples were cultured in parallel with Kit-M or Kit-I or without treatment as control for 7 days. Given are the mean frequencies ± standard deviations of (**a**) adaptive and innate immune cells and (**b**) IFNγ-producing cells as well as (**c**) degranulating cells. Statistical significance was tested by an independent-samples two-tailed *t*-test. (n) number of cases; significance is defined as “highly significant” in cases with *p*-values ≤ 0.005, “significant” with *p*-values ≤ 0.05, a “numerical trend” with *p*-values between 0.05 and 0.1, and “not significant” with *p*-values ≥ 0.1. Abbreviations of cell subpopulations are given in Table 1.

Comparing the efficacy of Kit-M- or Kit-I-pretreated vs. untreated AML samples studied in parallel after MLC (n = 10) on the provision of activated immune cells, we found not significantly increased frequencies of proliferating/non-naive memory cells and comparable frequencies of proliferating/non-naive memory cells (Figure 3a).

Comparing the efficacy of Kit-M- or Kit-I-pretreated samples after MLC vs. control on the provision of antigen-specific (SEB-stimulated) immune cells, we found significantly higher frequencies of IFNγ-producing cells after pretreatment in samples of healthy donors (n = 4) (e.g., %CIK_IFNγ_/CIK (Kit-I): 66.44 ± 7.25; %CIK_IFNγ_/CIK (Control): 30.76 ± 16.17; *p* < 0.001; %CIK_IFNγ_/CIK (Kit-M): 59.92 ± 24.93; %CIK_IFNγ_/CIK (Control): 30.76 ± 16.17; *p* < 0.001) as well as in AML samples (n = 10, e.g., %T_IFNγ_/T (Kit-I): 17.01 ± 7.43; %T_IFNγ/_T (Control): 6.65 ± 3.87; *p* < 0.001; %T_IFNγ/_T (Kit-M): 15.36 ± 6.00; %T_IFNγ/_T (Control): 6.65 ± 3.87; *p* < 0.001; Figure 3b). We also found numerical trends toward higher frequencies of TNFα-producing cells after pretreatment of healthy donors’ WB samples (n = 4, e.g., %T_TNFα/_T (Kit-I): 27.85 ± 7.72; %T_TNFα_/T (Control): 18.45 ± 5.96; *p* = 0.078; %T_TNFα_/T (Kit-M): 33.70 ± 16.37; %T_TNFα_/T (Control): 18.45 ± 5.96; *p* = 0.086) as well as in AML samples (n = 3, e.g., %CIK_TNFα_/CIK (Kit-I): 33.00 ± 6.40; %CIK_TNFα_/CIK (Control): 11.27 ± 9.82; *p* = 0.005; %CIK_TNFα_/CIK (Kit-M): 26.81 ± 17.51; %CIK_TNFα_/CIK (Control): 11.27 ± 9.82; *p* = 0.064. 

Comparing the efficacy of Kit-I- or Kit-M-pretreated vs. untreated cells after MLC in parallel analyses to produce activated/antigen-specific immune cells in AML (n = 10) or healthy samples (n = 4) after MLC, we found generally comparable frequencies of IFNγ-producing cells after Kit pretreatment in samples of healthy donors (e.g., %CIK_IFNγ_/CIK (Kit-I): 66.44 ± 7.25; %CIK_IFNγ_/CIK (Kit-M): 59.92 ± 24.93) as well as in AML samples (e.g., %T_IFNγ_/T (Kit-I): 17.01 ± 7.43; %T_IFNγ_/T (Kit-M): 15.36 ± 6.00; Figure 3b). We also found comparable frequencies of TNFα-producing cells after pretreatment in samples of healthy donors (n = 4, e.g., %T_TNFα_/T (Kit-I): 27.85 ± 7.72; %T_TNFα_/T (Kit-M): 33.70 ± 16.37) as well as in AML samples (n = 3, e.g., %CIK_TNFα_/CIK (Kit-I): 33.00 ± 6.40; %CIK_TNFα_/CIK (Kit-M): 26.81 ± 17.51. 

Comparing frequencies of degranulating CD107a-positive cells in Kit-I or Kit-M-pretreated vs. untreated cells after MLC in healthy donors’ WB samples (n = 6), we found comparable frequencies of degranulating cells after MLC (e.g., %B_107A_/B (Kit-I): 39.75 ± 19.86; %B_107A_/B (Kit-M): 31.68 ± 17.53; %B_107A_/B (Control): 17.75 ± 13.70; Figure 3c). Comparing the efficacy of Kit-M- vs. Kit-I-pretreated samples after MLC of healthy donors, we found, in some cases, not significantly higher frequencies of degranulating cells after pretreatment with Kit-M (e.g., %CIK_107A_/CIK (Kit-I): 29.50 ± 4.73; %CIK_107A_/CIK (Kit-M): 43.87 ± 18.41; Figure 3c). Comparing pretreated AML WB samples (n = 6) regarding the efficacy of Kit-I or Kit-M vs. control on the generation of degranulating cells, we found not significantly increased frequencies only after Kit-M pretreatment but not after Kit-I pretreatment (e.g., %CIK_107A_/CIK (Kit-I): 25.50 ± 11.08; %CIK_107A_/CIK (Kit-M): 33.33 ± 28.16; %CIK_107A_/CIK (Control): 25.71 ± 16.59; Figure 3c).

Antigen specificity was detected using SEB (in healthy) and WT1/PRAME-antigens in AML samples and showed, in principle, that antigen-specific reactions can be induced/are detected in healthy and AML samples, as shown before [10].

### 3.5. Effects of Kit-M- vs. Kit-I-Pretreated WB on Blast-Lytic Efficacy After MLC

**Figure 4:**
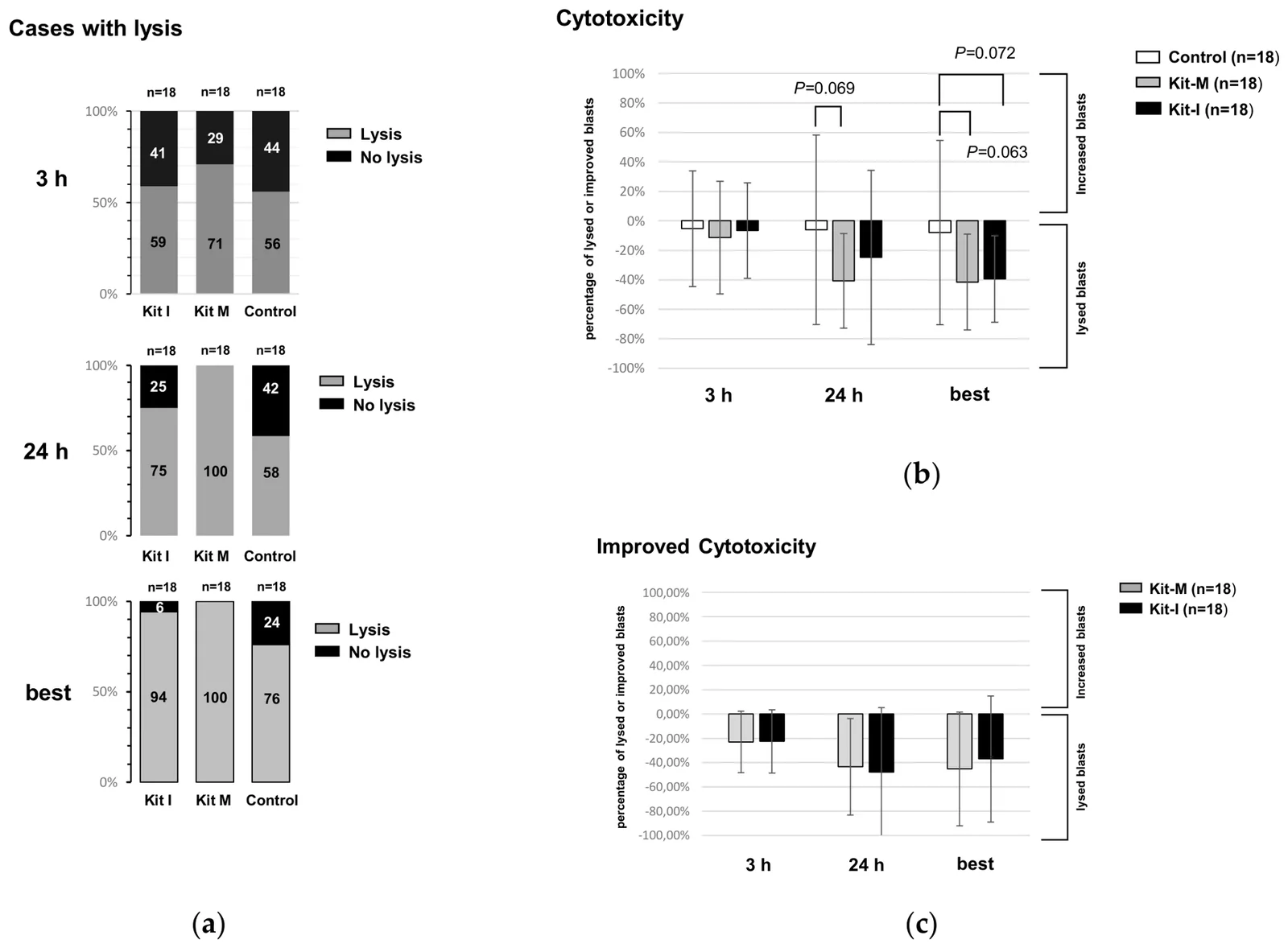
Effects of Kit-M- and Kit-I-treated AML WB on the antileukemic activity after MLC, detected via CTX. After MLC of Kit-M- or Kit-I-treated vs. untreated WB, these (‘immune effector’) cells were mixed with blast-containing MNCs (‘target cells’) and cultured for 3 h and 24 h. Given are the results after 3 h and 24 h and the best achieved lysis after either 3 h or 24 h of the incubation time of the effector and target cells. (**a**) The percentage of cases with lysis compared to the control group (with target and effector cells mixed shortly before the measurement (Control)) is shown. Given are the average ± standard deviations of (**b**) achieved cytotoxicity and (**c**) improved cytotoxicity. Statistical significance was tested by an independent-samples two-tailed *t*-test. (n) number of cases; significance is defined as “highly significant” in cases with *p*-values ≤ 0.005, “significant” with *p*-values ≤ 0.05, a “numerical trend” with *p*-values between 0.05 and 0.1, and “not significant” (n.s.) with *p*-values ≥ 0.1. Abbreviations of cell subpopulations are given in Table 1.

Pretreatment of leukemic WB with either Kit-I or Kit-M led to an increase in blast lysis after T-cell-enriched MLC compared to control, with more cases with lysis achieved after Kit-M pretreatment: A CTX fluorolysis assay showed blast lysis in 59% of Kit-I-pretreated cases after 3 h and in 75% after 24 h of co-incubation of effector and target cells. In Kit-M-pretreated samples, blast lysis was observed in 71% of cases after 3 h and in 100% after 24 h of co-incubation. After 24 h (Figure 4a), numerical trends toward increased blast lysis were observed in Kit-M and Kit-I-treated samples compared to control, selecting the best achieved lysis (*p* = 0.063 and *p* = 0.072, respectively). Comparing Kit-I vs. Kit-M in parallelly analyzed samples, a numerical trend toward improved lysis after 24 h was seen for Kit-M (*p* = 0.069; Figure 4b). Compared to control, improved cytotoxicity was shown for both Kit-I and Kit-M, with a slight advantage of Kit-M over Kit-I-pretreated WB (Figure 4c).

### 3.6. Correlation of the Relative Increase in Improved Blast Lysis in Kit-M- vs. Kit-I-Treated WB After MLC

**Figure 5:**
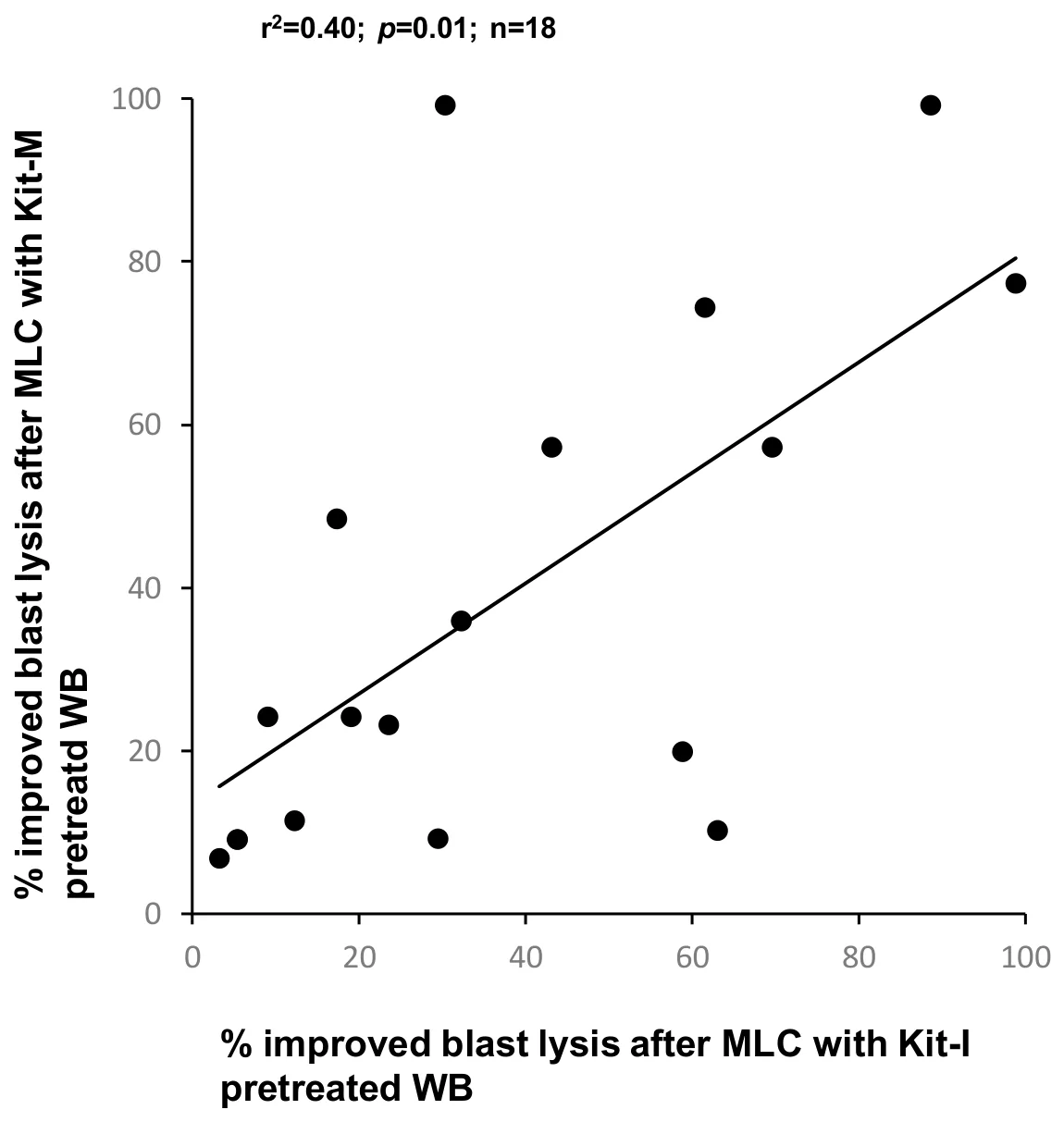
Correlation of the relative increase in improved blast lysis in Kit-M- vs. Kit-I-treated WB after MLC. Given are the coefficient of determination (r^2^) calculated by squaring the Pearson correlation coefficient, the *p*-value, and the number of cases (n); significance is defined as “significant” with *p*-values ≤ 0.05. Abbreviations of cell subpopulations are given in Table 2.

We found a significant positive correlation (*p* = 0.01; r^2^ = 0.40) between the relative increase in improved blast lysis after MLC with Kit-M-pretreated WB vs. improved blast lysis after MLC with Kit-I-pretreated WB. The y-intercept is at 13.41 (Figure 5).

### 3.7. Summarizing Conclusions

Both Kit-M and Kit-I produce DCs/DCleus and subsequently enhance (antileukemic) immune cell activation after MLC. Kit-M generated comparable or slightly higher frequencies of DCs/DCleus compared to Kit-I. We found higher frequencies of most leukemia-specific immune cells as well as elevated antileukemic cytotoxicity ex vivo. Compared to Kit-I, Kit-M-treated WB led to slightly faster effects on the provision of cytotoxic antileukemic activity after MLC as well as a higher relative increase in improved blast lysis.

## 4. Discussion

### 4.1. Immune Therapies and DC-Mediated Therapies to Overcome Immune Response

Currently, allogeneic stem cell transplantation remains the standard curative approach for acute myeloid leukemia (AML). Unfortunately, 30–60% of treated patients relapse, which is often caused by immune escape mechanisms [17,18]. Several immunotherapeutic strategies have the goal to restore effective immune control over leukemic cells, e.g., by reversing AML-induced immunosuppression using immune checkpoint inhibitors (PD1, CTLA4-expressing T cells) or combinations of antibodies addressing additional leukemic targets, thereby attenuating antileukemic immune responses, e.g., [5,19].

Given the limited immunogenicity of AML, a strategy that enhances immune responses may necessitate additional therapies addressing leukemic blasts directly [20].

DC-mediated immunotherapy offers a way that may avoid or overcome immune escape [13]. DCs are amongst the most potent mediators of the immune system. They are able to migrate to tissues in the whole body, present patient-specific blast antigens in a costimulatory manner to immune cells, and thus induce potent patient-specific antileukemic activity [12]. Disadvantages of approaches utilizing manipulated DCs loaded with antigens are the cost-, work-, and time-intensive production of DC or DCleus under GMP conditions, followed by a logistically challenging adoptive cell transfer to patients [21]. An approach based on treating patients directly with (clinically approved) immunomodulatory drugs (combined to Kits), aiming for patients to convert leukemic blasts to leukemia-derived DCs that activate the immune system, circumvents the ex vivo production of DCs or DCleus. With our previous data, we have shown that (using Kits (combined approved immune response modifiers) DCs generated from myeloid leukemic blasts (DCleus) in leukemic whole blood, simulating the in vivo situation, stimulate the immune system against various leukemic antigens [7,10]. Those DCs and DCleus can be generated both ex vivo, e.g., [7,10], and in vivo [11,13]. In vivo, it was possible to elicit leukemia-reactive immune responses in heavily pre-treated patients by applying GM-CSF and PGE1 (Kit-M). However, in these heavily pretreated end-stage patients, at least in some patients, this effect was transient. A rational combination with other immunomodulatory agents could be a way to improve this result [11,13]. We have previously shown that ex vivo several different agents can generate DCleus in WB of leukemic patients that subsequently lead to the induction of an antileukemic immune response in MLC [7]. One of these agents is Picibanil (or OK-432), which, in combination with granulocyte-macrophage colony-stimulating factor (GM-CSF; Kit-I), showed promising (dose-dependent for Picibanil (in Kit-I) or PGE1 (in Kit-M)) results ex vivo [12,22] as well as in vivo using a rat AML model [23]. Picibanil consists of deactivated Streptococcus pyogenes cells. It is known as a strong immunostimulant and sclerosing agent that is primarily used for the treatment of lymphangiomas [24]. In rats, Kit-I reduced regulatory T cells and induced antileukemic cells while showing no adverse effects [23].

### 4.2. Ex Vivo DC and DCleu Generation from Leukemic WB

At first, we confirmed that stimulation of uncultured leukemic WB with LAA/SEB leads to an increased formation of intracellularly cytokine-producing cells compared to healthy donors’ WB (Figure 1). This indicates that despite the disease, patients’ T cells are not exhausted but are still able to react to leukemic antigens [5,10]. We generated DCs and DCleus from leukemic WB using Kit-M and Kit-I (Figure 2). The use of WB instead of leukemic MNCs represents a more physiological environment, simulating in vivo conditions. Frequencies of DCs and DCleus were significantly higher in WB treated with either Kit compared to control. Importantly, the proliferation of leukemic blasts was induced by neither Kit-I nor by Kit-M-pretreated WB as stimulator cells compared to control without Kits. These findings confirm results that have been published and discussed before [6,7,10].

Both Kits provide a danger signal, thereby stimulating DC differentiation [25,26,27]. We found higher frequencies of mature DCs (DCmig/DC) in leukemic WB after using Kit-M compared to Kit-I, thereby partly confirming previous data that Kit-M (containing GM-CSF and PGE1) enhances the production of mature, migratory DCs [6,23]. These findings demonstrate that the mode of action of Kit-M or Kit-I in inducing the generation of DCs/DCleus might be different [22].

### 4.3. DC/DCleu-Induced Activation of Antigen/Leukemia-Specific Cells

We were able to confirm that IFNγ-producing immune cells were significantly increased after MLC using Kit-I- or Kit-M-pretreated samples (Figure 3b) [6]. Similar results were found for TNFα-producing cells. Since decreased IFNy production is a well-known immune inhibitory finding in leukemia, these observations might point to a mechanism to overcome immune escape ex vivo or in vivo: high frequencies of leukemia-specific cells were shown to be associated with longer remission-free intervals in patients after stem cell transplantation, pointing to an important, prognostically relevant significance of the presence of these cells [12,19,28].

Interestingly, we found highly significant differences in leukemia-specific CIK cells after MLC using Kit-I compared to using Kit-M. Degranulating, CD107a-positive cells have been described as antitumor-directed immune cells, with defined specificity after tumor antigen contact, e.g., [29,30].

We compared the frequencies of CD107a-positive degranulating cells in Kit-M- or Kit-I-pretreated cultures versus untreated controls after MLC (Figure 3c) and detected elevated frequencies of degranulating antigen-specific cells, thereby confirming preliminary data for Kit-M-mediated reactions for AML and healthy samples [10], whereas Kit-I-pretreated samples did not show differences for the AML cohort. Due to the low number of samples (and missing statistical significance), we cannot draw further conclusions, although differences seen for Kit-M- vs. Kit-I-pretreated AML samples again might point to different modes of action of the two kits.

### 4.4. Mediation of Antileukemic Cytotoxicity

We found higher and improved lytic activity against leukemic blasts induced by Kit-M as well as by Kit-I, comparing effects of Kit-M- vs. Kit-I-pretreated WB on blast-lytic efficacy after MLC. We found slightly higher cytotoxicity for Kit-M and Kit- I-pretreated cells after 3 h vs. 24 h of co-incubation of target and effector cells (Figure 4). This might be attributed to different cytotoxic modes of action of immune-reactive cells, as shown before [12]: the perforin/granzyme pathway is much faster than the late and slower-acting Fas/FasL pathway [31]. The faster-acting blast-lytic activity induced by Kit-M- vs. Kit-I-pretreated WB might point to different mechanisms elicited by Kit-M vs. Kit-I upon activation of immune-active cells and subsequent cytotoxic activities against leukemic blasts.

### 4.5. Correlation of Cytotoxicity of Kit-M- vs. Kit-I-Pretreated WB

The relative cytotoxicity in Kit-M- vs. Kit-I-treated WB after MLC showed a strong and significant correlation (Figure 5), although the regression line might indicate a stronger Kit-M- vs. Kit-I-mediated blast kill. This is consistent with previous findings in Kit-M-treated WB samples or in syngeneic rats, where Kit-M induced a stronger reduction of blasts in blood compared to Kit-I [6,7,23].

This finding might again point to different functional mechanisms by which the two kits elicit their effect.

Our data might point to subgroups of patients who might be either more responsive to treatment with Kit-M or with Kit-I or a combination of both. Further research is necessary to elucidate the underlying mechanism as well as possible predictors for that response to be able to treat patients with the suitable agent.

## 5. Conclusions

In summary, our study demonstrates that both Kit-M and Kit-I enhance (leukemia-specific) immune cell activation and antileukemic cytotoxicity via a DC/DCleu-mediated mechanism.

Our results might indicate that Kit-M and Kit-I operate through distinct mechanisms and that some patients’ WB elicits stronger blast lysis after MLC when previously incubated using Kit-M compared to Kit-I, and vice versa. With respect to clinical application, our data might offer the possibility to enhance (Kit-mediated) treatment by combining approved immunomodulatory agents and identifying the Kit with superior mediation of antileukemic reactivity for individual patients.

## 6. Limitations

Limitations and confounding factors might be the differing ages in healthy vs. patient blood donors (31.5 vs. 57.9 years) and the small sample sizes for some comparisons. In consequence, some comparisons yielded numerical trends to distinguish groups (instead of statistically significant differences). These findings require confirmation in larger, adequately powered prospective studies.

## Figures and Tables

**Table 1:** Cells and cell subsets as evaluated by flow cytometry.

Cell Type	Name of Subgroups	Abbreviation of Subgroups	Surface Marker	Referred to	Abbreviation	References
Subtypes of blasts and DCs						
Blast cells	Leukemic blasts	Bla	Bla^+^ (e.g., CD34^+^, CD177^+^)	WB	Bla/WB	[7]
	Proliferating blasts	Blaprol-CD71	Bla^+^DC^−^CD71^+^	Bla	Blaprol-CD71/Bla	[7]
	Proliferating blasts	Blaprol-IPO38	Bla^+^DC^−^IPO38^+^	Bla	Blaprol-IPO38/Bla	[7]
Dendritic cells	Dendritic cells	DC	DC^+^ (CD80^+^CD206^+^)	WB	DC/cells	[7]
	Leukemia-derived DC	DCleu	DC^+^Bla^+^	WB	DCleu/cells	[7]
				Bla	DCleu/Bla	[7]
	Mature DC	DCmig	DC^+^CD197^+^	WB	DCmig/cells	[7]
	Mature DC	DCmig	DC^+^CD197^+^	DC	DCmig/DC	[7]
	Mature DCleu	DCmig-leu	DC^+^CD197^+^Bla^+^	WB	DCmig-leu/cells	[7]
				DC	DCmig-leu/DC	[7]
				DCleu	DCmig-leu/DCleu	[7]
Subtypes of immune-reactive cells						
	CD4^+^-coexpressing T cells	T4+	CD3^+^CD4^+^	T	T4+/T	[6]
	Non-naive T cells	Tnn	CD3^+^CD45RO^+^	T	Tnn/T	[6]
	Central (memory) T cells	Tcm	CD3^+^CD45RO^+^CD197^+^	T	Tcm/T	[6]
	Effector (memory) T cells	Tem	CD3^+^CD45RO^+^CD197^−^	Tem	Tem/T	[6]
	Proliferating T cells—early	Tprol-early	CD3^+^CD69+	T	Tprol-early/T	[6]
	Proliferating T cells—late	Tprol-late	CD3^+^CD71^+^	T	Tprol-late/T	[6]
Subtypes of different intracellularly IFNy- or TNFa-producing cells						
TT cells	CD3^+^ pan T cells	T_IFNy/TNFa_	IFNy^+^/TNFa^+^CD3^+^	T	T_IFNy/TNFa_/T	[6]
	CD4^+^-coexpressing T cells	T4+_IFNy/TNFa_	IFNy^+^/TNFa^+^CD3^+^CD4^+^	T4+	T4^+^_IFNy/TNFa_/T4^+^	[6]
	CD8^+^-coexpressing T cells	T4−_IFNy/TNFa_	IFNy^+^/TNFa^+^CD3^+^CD8^+^	T4−	T4-_IFNy/TNFa_/T4^−^	[6]
	Non-naive T cells	Tnn_IFNy/TNFa_	IFNy^+^/TNFa^+^CD3^+^CD45RO+	Tnn	Tnn_IFNy/TNFa_/Tnn	[6]
	Effector (memory) T cells	Tem_IFNy/TNFa_/Tem	IFNy^+^/TNFa^+^CD3^+^CD45RO^+^CD197^−^	Tem	Tem_IFNy/TNFa_/Tem	[6]
	Central (memory) T cells	Tcm_IFNy/TNFa_	IFNy^+^/TNFa^+^CD3^+^CD45RO^+^CD197^+^	Tcm	Tcm_IFNy/TNFa_/Tcm	[6]
NK cells	CD3^−^CD56^+^NK cells	NK_IFNy/TNFa_	IFNy^+^/TNFa^+^CD3^−^CD56^+^	NK	NK_IFNy/TNFa_/NK	[6]
CIK cells	CD3^+^CD56^+^ CIK cells	CIK_IFNy/TNFa_	IFNy^+^/TNFa^+^CD3^+^CD56^+^	CIK	CIK_IFNy/TNFa_/CIK	[6]
Subtypes of different degranulating (CD107a^+^) cells						
T cells	CD3^+^ pan T cells	T_107a_	CD107a^+^CD3^+^	T	T_107a_/T	[10,14]
	CD4^+^-coexpressing T cells	T4+_107a_	CD107a+CD3^+^CD4^+^	T	T4+_107a_/T	[10]
	CD8^+^-coexpressing T cells	T4−_107a_	CD107a+CD3^+^CD8^+^	T	T4−_107a_/T	[10]
	Non-naive T cells	Tnon-naive_107a_	CD107a^+^CD3^+^CD45RO+	Tnn	Tnn_107a_/Tnn	[10,14]
	Effector (memory) T cells	Tem_107a_	CD107a^+^CD3^+^CD45RO^+^CD197^−^	Tem	Tem_107a_/Tem	[14]
	Central (memory) T cells	Tcm_107a_	CD107a^+^CD3^+^CD45RO^+^CD197^+^	Tcm	Tcm_107a_/Tcm	[14]
B cells	CD19^+^	B_107a_	CD107a^+^CD19^+^	B	B_107a_/B	[14]
NK cells	CD3^−^CD56^+^NK cells	NK_107a_	CD107a^+^CD3^−^CD56^+^	NK	NK_107a_/NK	[10,14]
CIK cells	CD3^+^CD56^+^CIK cells	CIK_107a_	CD107a^+^CD3^+^CD56^+^	CIK	CIK_107a_/CIK	[10,14]

**Table 2:** Patients’ characteristics.

Patient No.	Sex	Age at dgn	FAB Type	Stage	Blasts in PB (%) *	Blast Phenotype (CD)	ELN-Risk Stratification	Response to Induction Chemotherapy	Conducted Experiments		
									In Uncultured WB	After Kit-I Treatment	After Kit-M Treatment
**AML**											
P1426	f	59	sAML/M5	dgn	30	13, 33, **34**, **117**		response		CTX	CTX
P1430	m	79	pAML	dgn	70	13, 38, **34**, **117**	intermediate	no response		CTX	CTX
P1432	m	34	pAML/M4/M5	dgn	80	13, 33, 116, 4	favorable	response		CTX	CTX
P1447	m	21	pAML/M5	dgn	63	33, **65**	intermediate	response		CTX	CTX
P1463 (2)	f	58	sAML (MDS)	dgn	30	33, 13, 56	adverse	relapse		CTX	CTX
P1471	m	39	pAML/M1	dgn	69	13, **34**, **117**	adverse	no response		CTX	CTX
P1472	f	33	pAML/M2	dgn	30	13, 33, **34**, **117**	favorable	response		CTX	CTX
P1480	m	66	sAML	dgn	20	13, 33, **117**	adverse	no response		CTX	CTX
P1483	m	77	AML/M5	dgn	60	13, 33, **34**, 64	adverse			CTX	CTX
P1509	m	60	pAML/M2	dgn	48	13, 33, 34, **65**, **117**	favorable	response	FC, DC-FC, INCYT_INFy_, INCYT_TNFa_, DEG	FC, DC-FC, INCYT_INFy_, INCYT_TNFa_, DEG, CTX	FC, DC-FC, INCYT_INFy_, INCYT_TNFa_, DEG
P1512	f	80	pAML	dgn	40	13, **34**, 117	adverse	no response	FC, DC-FC, INCYT_INFy_, INCYT_TNFa_, DEG	FC, DC-FC, INCYT_INFy_, INCYT_TNFa_, DEG	
P1514	m	68	sAML	dgn	51	33, 56, **117**	favorable	no response	FC, DC-FC, INCYT_INFy_, INCYT_TNFa_, DEG	FC, DC-FC, DEG	FC, DC-FC, DEG
P1516	f	52	dAML	dgn	58	**34**, **117**, 13, 56, 7	adverse	no response	FC, DC-FC, INCYT_INFy_, INCYT_TNFa_, DEG	FC, DC-FC	FC, DC-FC, INCYT_INFy_, INCYT_TNFa_
P1518	f	83	pAML/M2	dgn	72	14, 15, **34**, **65**	favorable	no response	FC, DC-FC, INCYT_INFy_, INCYT_TNFa_	FC, DC-FC, INCYT_INFy_, INCYT_TNFa_	FC, DC-FC, INCYT_INFy_, INCYT_TNFa_
P1525	m	77	pAML/M1	dgn	78	13, 15, 33, **34**, **117**	intermediate		CSA_INFy_	CSA_INFy_	CSA_INFy_
P1526	f	74	pAML	dgn	61	15, 33, **34**, 56, 65, **117**	favorable		FC, DC-FC, CSA_INFy_, DEG	FC, DC-FC, CSA_INFy_, DEG	FC, DC-FC, CSA_INFy_, DEG
P1527	m	42	pAML/M2	dgn	28	7, 13, 15, 33, **34**, 65, **117**	intermediate	response	FC, DC-FC, INCYT_INFy_, INCYT_TNFa_, DEG	FC, DC-FC, INCYT_INFy_, INCYT_TNFa_, DEG, CTX	FC, DC-FC, DEG, CTX
P1536	m	61	pAML/M5	dgn	73	14, **34**, 56	favorable	no response		CSA_INFy_, CTX	CSA_INFy_, CTX
P1568	m	29	pAML	dgn	79	10, 13, 33, **34**	adverse	response		CSA_INFy_	CSA_INFy_, CTX
P1570	f	36	pAML	dgn	33	7, 13, 14, 33, **34**, **117**	favorable	response		CSA_INFy_, CTX	CSA_INFy_, CTX
P1572	f	63	sAML	dgn	12	13, 33, **34**, 65, **117**	adverse	response	INCYT_INFy_, INCYT_TNFa_, DEG	CSA_INFy_, DEG	DC-FC, CSA_INFy_, DEG
P1386	m	57	sAML	relapse	80	**34**, **117**, 65, 33, 13				CTX	CTX
P1434	f	61	sAML	relapse	59	13, 33, **34**, 56, 64, **117**				CTX	CTX
P1457	m	63	sAML	relapse	12	12, 34, **117**				CTX	CTX
P1497	m	74	sAML	relapse	59	13, **34**, 65, **117**			FC, DC-FC, INCYT_INFy_, INCYT_TNFa_	FC, DC-FC, INCYT_INFy_, INCYT_TNFa_, CTX	CTX
P1521	m	56	pAML/M4	relapse	72	13, 15, 33, **34**, 65, **117**				CSA_INFy_	CSA_INFy_
P1522	m	47	sAML	relapse	55	13, **34**, 71, **117**			FC, DC-FC, INCYT_INFy_, INCYT_TNFa_, DEG	FC, DC-FC, DEG	FC, DC-FC, DEG
P1387	m	73	AML/M4	relapse after SCT	85	**117**, 33, 13				CTX	CTX
**HEALTHY**											
P1510	m	22							DC-FC, INCYT_INFy_, INCYT_TNFa,_ DEG	DC-FC, INCYT_INFy_, INCYT_TNFa,_ DEG	DC-FC, INCYT_INFy_, INCYT_TNFa_, DEG
P1513	m	27							DC-FC, INCY_INFy_, INCYT_TNFa_, DEG	DC-FC, INCYT_INFy_, INCYT_TNFa_, DEG	DC-FC, DEG, INCYT_INFy_, INCYT_TNFa_, DEG
P1517	m	39							DC-FC, INCYT_INFy_, INCYT_TNFa_, DEG	DC-FC, INCYT_INFy_, INCYT_TNFa_, DEG	DC-FC, INCYT_INFy_, INCYT_TNFa_, DEG
P1523	m	17							DC-FC, INCYT_INFy_, INCYT_TNFa_, DEG	DC-FC, INCYT_INFy_, INCYT_TNFa_, DEG	DC-FC, INCY_TINFy_, INCYT_TNFa_, DEG
P1566	f	54							FC, DC-FC, INCY_INFy_, INCYT_TNFa_, DEG	DEG	FC, DC-FC, INCY_TINFy_, INCYT_TNFa_, DEG
P1579	m	30							FC, DC-FC, INCYT_INFy_, INCYT_TNFa_, DEG	DEG	FC, DC-FC, INCYT_INFy_, INCYT_TNFa_, DEG

Legend: f: female; m: male; FAB type: French–American–British classification of AML; pAML: primary AML, sAML: secondary AML; M1–5: subtypes of the disease under the FAB classification; ELN: European Leukemia Network; CD: cluster of differentiation; bold: antibody used for expression analyses; dgn: diagnosis; FC: flow cytometric analyses; DC-FC: flow cytometric analyses after dendritic cell culture; DEG: degranulation assay; INCYT: intracellular cytokine assay; CSA: Cytokine Secretion Assay; CTX: cytotoxicity fluorolysis assay; * at day of sampling, measured by flow cytometry. The bold formatting is intended to highlight the relevant values for clarify and should be retained.

## Data Availability

Data are contained within the article.

## Supplementary Materials

The following supporting information can be downloaded at https://www.mdpi.com/article/10.3390/cancers18172729/s1, Figure S1: DC/DCleu generation using Kit-M or Kit-I (1) to generate DCs/DCleus, followed by Kit-mediated antileukemic processes (as shown by functional leukemia-specific or cytotoxicity assays) after T-cell-enriched MLC [2,3].

## Abbreviations

The following abbreviations are used in this manuscript:

AML	Acute Myeloid Leukemia
DC	Dendritic Cells
DCC	Dendritic Cell Culture
DEG	Degranulation Assay
CSA	Cytokine Secretion Assay
CTX	Cytotoxicity Assay
ELN	European LeukemiaNet
GM-CSF	Granulocyte-Macrophage Colony-Stimulating Factor
IFNγ	Interferon Gamma
INCYT	Intracellular Cytokine Assay
LAA	Leukemia Associated Antigens
MLC	Mixed Lymphocyte Culture
moDC	Monocyte-Derived DC
PGE-1	Prostaglandin E1
SCT	Stem Cell Transplantations
TNFα	Tumor Necrosis Factor alpha
WB	Whole Blood
