# Supplementary material for: Comparative Evaluation of Kit-M (GM-CSF and PGE-1) and Kit-I (GM-CSF and Picibanil) for the Generation of DCs/DCleus and Their Impact on Subsequent Antileukemic Immune Cell Activation Ex Vivo
Source: Cancers (Basel). 2026 Aug 23;18(17):2729. doi: 10.3390/cancers18172729 (PMC13565580; doi:10.3390/cancers18172729)
Supplement: Supplementary file 1 [file cancers-18-02729-s001.zip › cancers-4452726-supplementary.pdf]

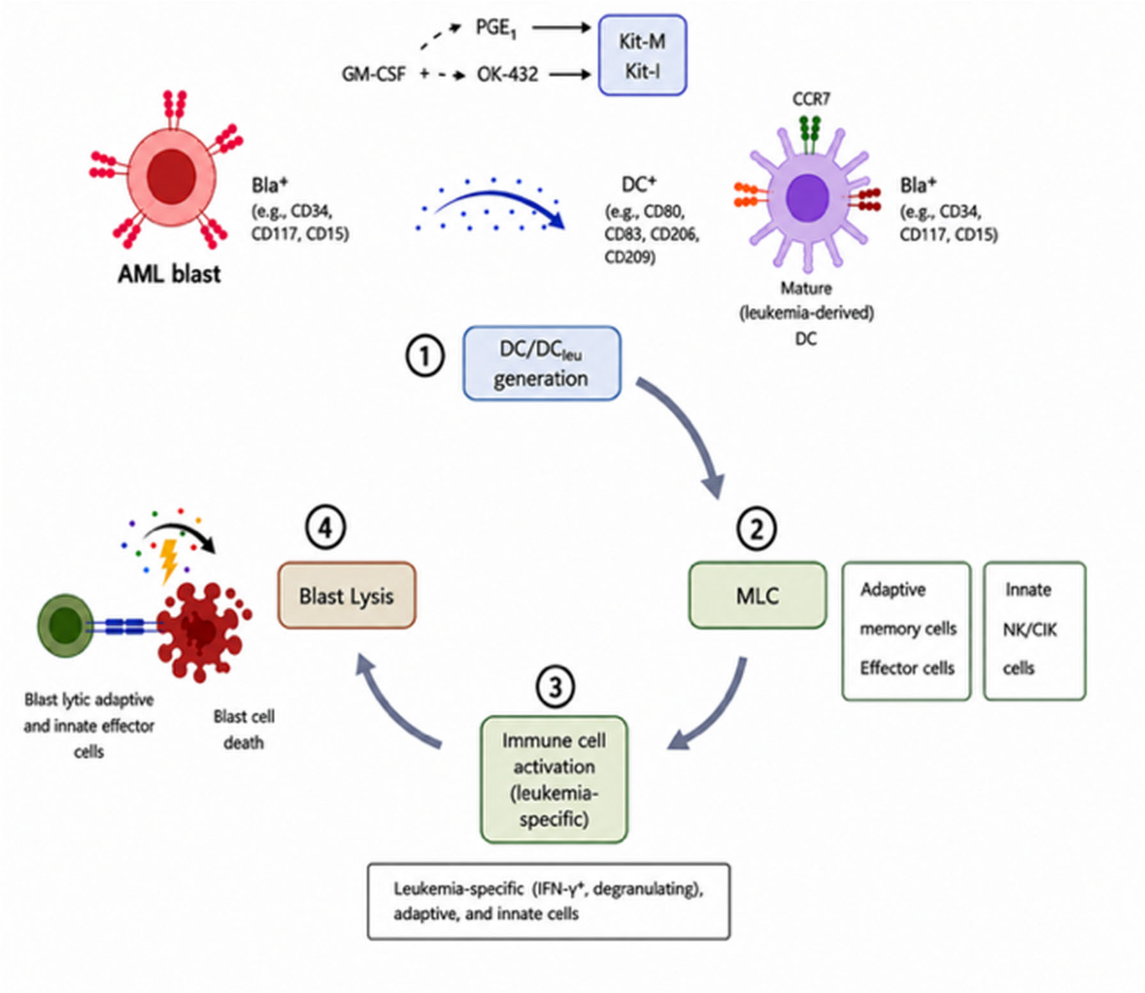

Supplementary Figure S1. DC/DCleu generation using Kit-M or Kit-I (1) to generate DC/DCleu, followed by Kit-mediated antileukemic processes (as shown by functional leukemia-specific or cytotoxicity assays) after T-cell-enriched MLC [2,3].
